# Automatic irrigation system with a fiber-optic pressure sensor regulating intrapelvic pressure for flexible ureteroscopy

**DOI:** 10.1038/s41598-023-47373-5

**Published:** 2023-12-21

**Authors:** Takashi Yoshida, Noriko Tsuruoka, Yoichi Haga, Hidefumi Kinoshita, Sang-Seok Lee, Tadao Matsunaga

**Affiliations:** 1 Graduate School of Engineering, Tottori University, Tottori, Japan; 2 Department of Urology and Andrology, Kansai Medical University, Osaka, Japan; 3 Graduate School of Engineering, Tohoku University, Sendai, Japan; 4 Graduate School of Biomedical Engineering, Tohoku University, Sendai, Japan; 5 Advanced Mechanical and Electronic System Research Center, Faculty of Engineering, Tottori University, 4-101 Koyama Minami, Tottori, 680-8552 Japan

**Keywords:** Urology, Engineering

## Abstract

Increased intrapelvic pressure (IPP) due to irrigation during flexible ureteroscopy (f-URS) can pose a risk of postoperative severe urinary tract infection associated with pyelovenous backflow. An automatic regulation system for maintaining safe IPP levels could enable surgeons to perform f-URS safely without postoperative complications. This study aimed to assess the measurement accuracy of an ultra-miniature fiber-optic pressure sensor incorporated into a small-caliper ureteroscope for assessing IPP and to develop an automatic irrigation system linked to this sensor. A porcine kidney was used for the ex vivo experiment. The nephrostomy catheter, connected to the conventional pressure transducer, was placed on the renal pelvis to evaluate the actual IPP (a-IPP). For measuring IPP using the fiber-optic pressure sensor (fo-IPP) built into the f-URS, a diaphragm pressure sensor of Φ250 μm was used. To establish an irrigation system, the optimal proportional–integral–derivative (PID) controller was explored to accurately adjust the irrigation pump flow rate. A high correlation between a-IPP and fo-IPP was confirmed across irrigation pressure values of 60–180 mbar (all, r ≥ 0.7, p < 0.001). When performing bolus irrigation, although fo-IPP showed relatively a higher peak value than a-IPP, the response time of fo-IPP was equivalent to that of a-IPP. After PID parameter optimization, our automatic irrigation system based on fo-IPP smoothly and accurately regulated the intended IPP set in the 5–20 mmHg range without overshooting. We successfully developed and demonstrated an automatic irrigation system regulating IPP based on the PID controller for f-URS, utilizing a fiber-optic pressure sensor. Further research, including in vivo studies, will be needed to assess clinical feasibility.

## Introduction

In daily clinical practice in the field of urology, flexible ureteroscopy (f-URS) is a widely accepted procedure for the treatment of kidney stones^1,2^. Adequate irrigation flow is essential to maintain clear visibility and a wide surgical field during f-URS. However, increased intrapelvic pressure (IPP) can be associated with pyelovenous or pyelosinus backflow, increasing the risk of severe postoperative urinary tract infection and sepsis^3,4^. The CROES URS Global Study reported instances of Clavien grade III (requiring surgical or radiological intervention), IV (life-threatening complication), and V (death) events due to fever, urinary infection, and sepsis, despite the relatively low overall complication rates of 7.4% with 874 out of 11,885 patients^5^. Various irrigation devices, such as gravity-driven bags, pressurized irrigation bags, hand-operated irrigation pumps, and continuous irrigation systems^6^, are available and selected for use at the surgeon’s discretion based on their experience. Bolus irrigation, an on-demand force irrigation, is also commonly used to achieve clear visibility when the surgical field is disturbed by bleeding or floating stone dust. However, this procedure poses a risk of a rupturing the collecting system due to extremely high IPP^4^. To date, no consensus on a standardized method for irrigation with a safe level of IPP has been established to prevent postoperative complications.

In laparoscopic surgery, the automatic pressure-regulating pneumoperitoneum device enables the operator to create an adequate and safe surgical field, allowing them to focus solely on the surgical procedure^7^. In the context of f-URS, previous studies have evaluated IPP using a ureteral catheter, nephrostomy tube, or pressure wire^8–14^. Recently, a new disposable 9.5Fr f-URS with a pressure sensor, LithoVue™ Elite, has been introduced, providing real-time IPP information to surgeons. However, an irrigation system regulated based on IPP values, facilitating standardized surgical settings and prevention of postoperative complications, has yet to be established. Thus, we sought to develop a comprehensive irrigation system to address this gap.

The objective of this study was to assess whether our previously developed ultra-miniature fiber-optic pressure sensor, which can be built into a ureteroscope without compromising flexibility, could accurately measure the actual IPP (a-IPP). Furthermore, we constructed an automatic irrigation system based on a proportional–integral–derivative (PID) controller, a type of control system that uses feedback to continuously adjust the output of a process or system to match a desired setpoint.

## Materials and methods

### Experimental setting

**Figure 1:**
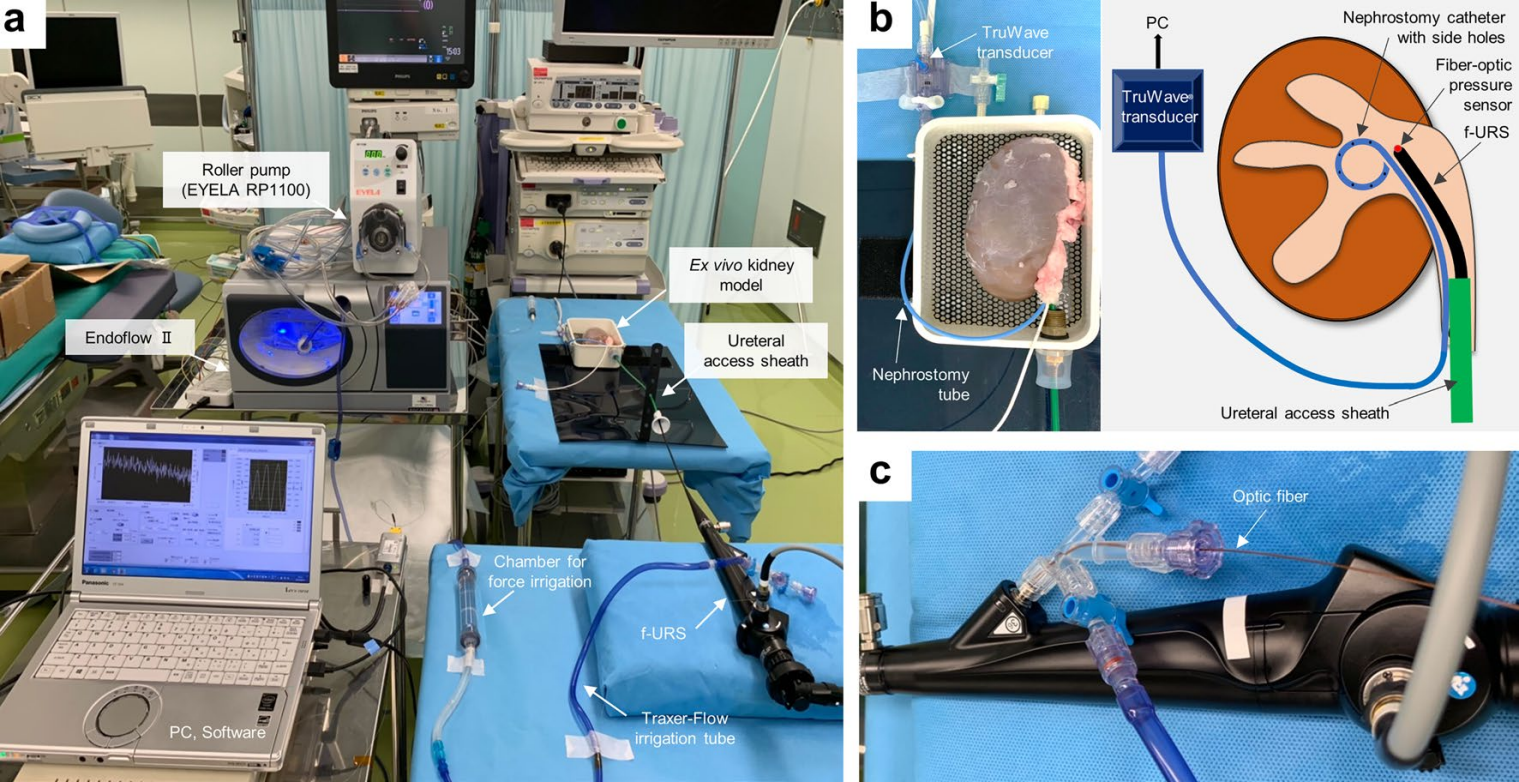
The experimental setting. (**a**) The entire setting. (**b**) Details of the ex vivo kidney model and their scheme. (**c**) A flexible ureteroscope (f-URS) with optic fiber.

The entire experimental setting of this study is shown in Fig. 1a. The continuous irrigation pump (Endoflow^®^ II, Rocamed, Avenue Albert II, Monaco), which utilizes air compression to maintain a consistent irrigation volume through a pressure-controlled system, was used for irrigation with natural saline. A fresh cadaveric porcine kidney (weight: 241.3 g, maximum length: 12.8 cm) (Maeda pork Co., Ltd, Hyogo, Japan) with a T-box was used as an ex vivo kidney model (Fig. 1b). The T-box is an experimental model we developed previously for the objective evaluation of surgical instruments and devices in retrograde intrarenal surgery^15,16^. Additionally, it facilitates the setup of ex vivo kidney models, ensuring consistent experimental conditions. In this study, no animal ethics committee approval was necessary since slaughterhouse waste material was used. An 8.3-Fr nephrostomy catheter (JINRO™, Boston Scientific, Massachusetts, USA) and a 10/12-Fr ureteral assess sheath (UAS; Proxis^®^, BD, Covington, USA) were tightly secured to the ureteropelvic junction. The tip of the nephrostomy catheter and a 7.95-Fr f-URS (URF-P6^®^, Olympus, Tokyo, Japan) were placed on the center of the renal pelvis.

### Devices for measuring IPP

To evaluate a-IPP, a pressure transducer (TruWave^®^, Edwards Lifesciences, Irvine, US) was connected to the end of an 8.3-Fr nephrostomy catheter filled with natural saline (Fig. 1b). The TruWave is widely used for clinical and bench investigations to study biological pressure, with a sensitivity of 5.0 µV/V/mmHg ± 1% and an accuracy of ± 1.5% of reading or ± 1 mmHg^17,18^. The output voltage from the TruWave, which is proportional to pressure, was measured using a myDAQ device (National Instruments, Austin, US) connected to the PC.

**Figure 2:**
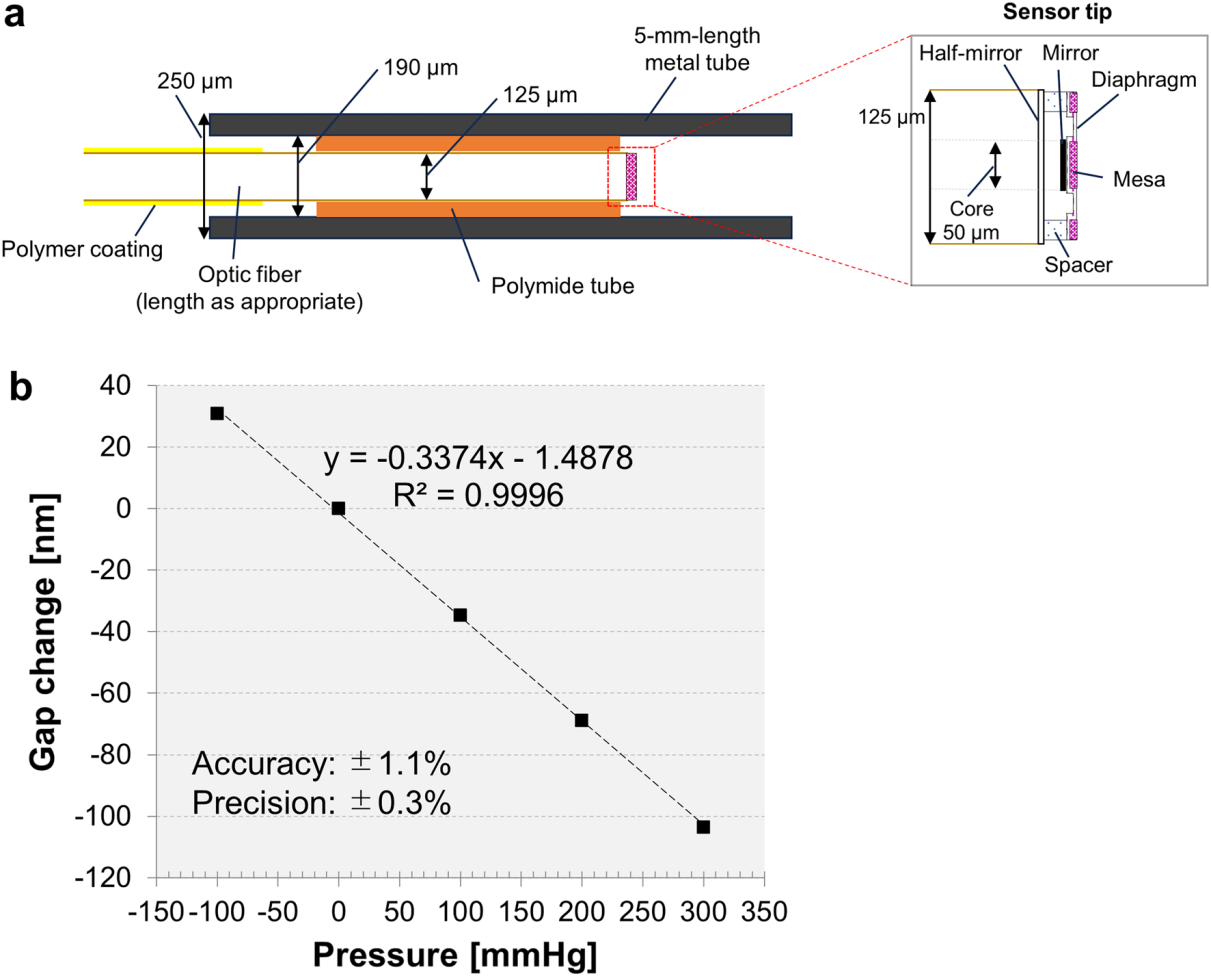
Characteristics of the fiber-optic pressure sensor used in this study. (**a**) The structure of the fiber-optic sensor. (**b**) The correlation between the gap change of the total reflection mirror and the half mirror, and the applied pressures.

For measuring IPP using a fiber-optic pressure sensor (fo-IPP), we used our developed fiber-optic pressure sensor, which was placed on a working channel of the f-URS (Fig. 1b,c). Briefly, this sensor, fabricated by Micro Electro Mechanical Systems (MEMS) technology, has a total reflection mirror formed on the diaphragm and a half-mirror formed at the end of the optic fiber (Fig. 2a)^19,20^. A multi-mode optical fiber with a core diameter of 50 μm and an outer diameter of 125 μm, was used. The sensor was enclosed in a 5-mm-length metal pipe with an outer diameter of 250 μm and connected to the optic fiber (Fig. 2a). The tip of the sensor was placed at 2 mm inside the tip of the ureteroscope working channel. A Fabry–Perot interferometer was used to measure the deflection of the diaphragm derived from the pressure. The low temporal coherence of the white light-emitting diode, passing through the optic fiber to the sensor element, is modulated interferometrically based on the gap change between the total reflection mirror and the half mirror. The light reflected from the sensor passes back via the same fiber and is detected by a spectrometer^20^. The spectrometer was connected to the PC, and the pressure values were calculated using our self-developed LabView software. In this study, the fiber-optic pressure sensor with a sensitivity of − 0.3374 nm/mmHg and an accuracy of ± 1.1% was used (Fig. 2b).

### Evaluation of the performance of a fiber-optic pressure sensor

To evaluate the measurement performance of the fiber-optic pressure sensor, the correlations between a-IPP and fo-IPP were assessed. The zero points of both pressure sensors were calibrated with the renal pelvis completely collapsed. Irrigation for the ex vivo kidney model was performed using the continuous irrigation pump (Endoflow^®^ II) with 37 °C natural saline at irrigation pressures of 60, 100, 120, and 180 mbar (1 mbar is equivalent to 0.75006 mmHg) through the f-URS working channel for approximately 180 s (s) from the start to end. Throughout irrigation, a-IPP and fo-IPP were simultaneously recorded, confirming the rise of pressure from the 0 points, plateau, and decline back to the 0 points. We assessed the accuracy and precision of the fiber-optic pressure sensor against the TruWave during the plateau phase where we could evaluate IPP fluctuations of each sensor at the same pressure values.

An additional experiment was conducted to evaluate the measurement accuracy and pressure dynamics of the fiber-optic pressure sensor during bolus irrigation, in comparison to those of the TruWave. Continuous flow irrigation was maintained at a pressure of 100 mbar, and several instances of forced irrigation for approximately one second each were performed using the hand-pump chamber of the irrigation tube. Both a-IPP and fo-IPP were simultaneously recorded, and the response time (defined as the time from start to peak) and the peak range (defined as the difference in IPP value from baseline to peak) of each sensor were assessed.

### Construction and evaluation of an automatic irrigation system

#### System design

**Figure 3:**
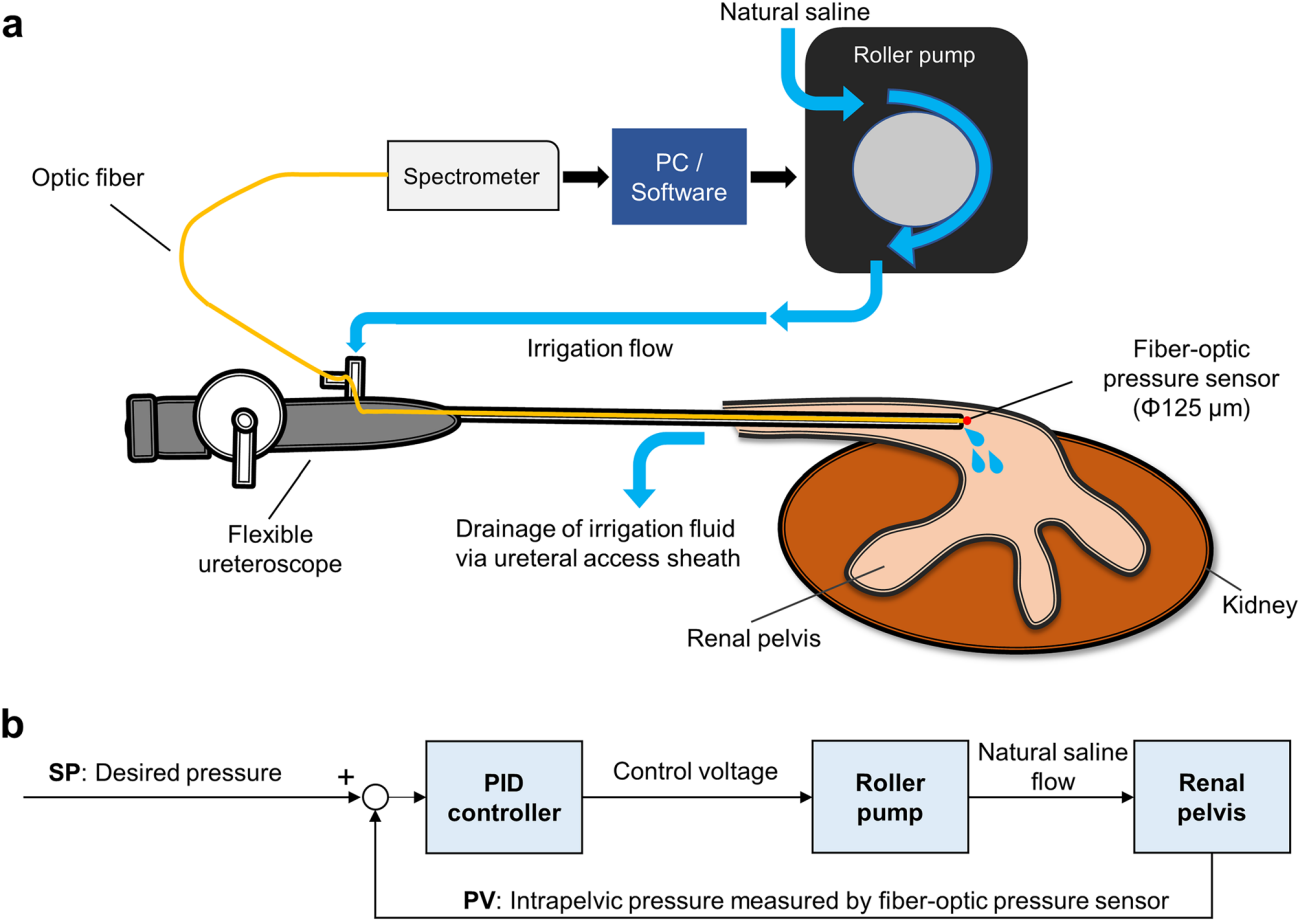
The concept of automatic irrigation system. (**a**) Scheme of the entire system. (**b**) The PID control block scheme. *PC* personal computer, *PID* proportional integral derivative, *PV* process variable, *SP* set point.

An overview of the concept of the automatic irrigation system is shown in Fig. 3a. The entire control process of real-time automatic irrigation is based on the control of the pressure derived from the fiber-optic pressure sensor via the spectrometer as input, and the control of the voltage to the DC motor of a roller pump (EYELA RP-1100, TOKYO RIKAKIKAI CO, LTD, Tokyo, Japan) as the output. Based on the value of fo-IPP, a PID controller is then set and subsequently optimized to maintain the pump flow rate at a specified and desired value.

#### Modeling of the irrigation system controller

The PID control block scheme of the fabricated system is shown in Fig. 3b. A PID controller consists of three components that are adjusted based on the difference between a set point (SP) and a measured process variable (PV).

1 $$ e\left( t \right) = SP \, - \, PV. $$

The output of a PID controller (u(t)) is calculated using the sum of the Proportional, Integral, and Derivative terms where KP, KI, and KD are constants that can be adjusted to fine-tune the performance of the controller.

2 $$u\left(t\right)=\mathrm{ KP}e\left(t\right)+\mathrm{KI}\int \limits_{0}^{t}e\left(t\right)dt+\mathrm{KD}\frac{de\left(t\right)}{dt}.$$

The proportional coefficient KP can adjust the deviation and improve the control sensitivity promptly, but it cannot eliminate the steady-state error of the PID control system. The integral coefficient KI can eliminate the steady-state error of the system. The differential coefficient KD can enhance the response speed of the system and reduce oscillation, but excessively large integral and differential coefficients might impact the stability of the system. Therefore, optimizing the parameters of the PID controller and identifying its optimal combination can improve the control effect of the PID controller.

In the fabricated irrigation system, a desired pressure value was inputted as SP, and the rotation speed of the roller pump was calculated as the control signal u(t). The rotation speed of the roller pump was controlled by inputting a voltage signal corresponding to the desired rotation speed. Natural saline was irrigated by the roller pump into the renal pelvis at a calculated rotation speed, which was then converted into IPP. The pressure value (PV) was measured by the fiber-optic pressure sensor. The control voltage was applied to the roller pump through myDAQ. This feedback system was constructed using the PID VI of LabView software.

**Figure 4:**
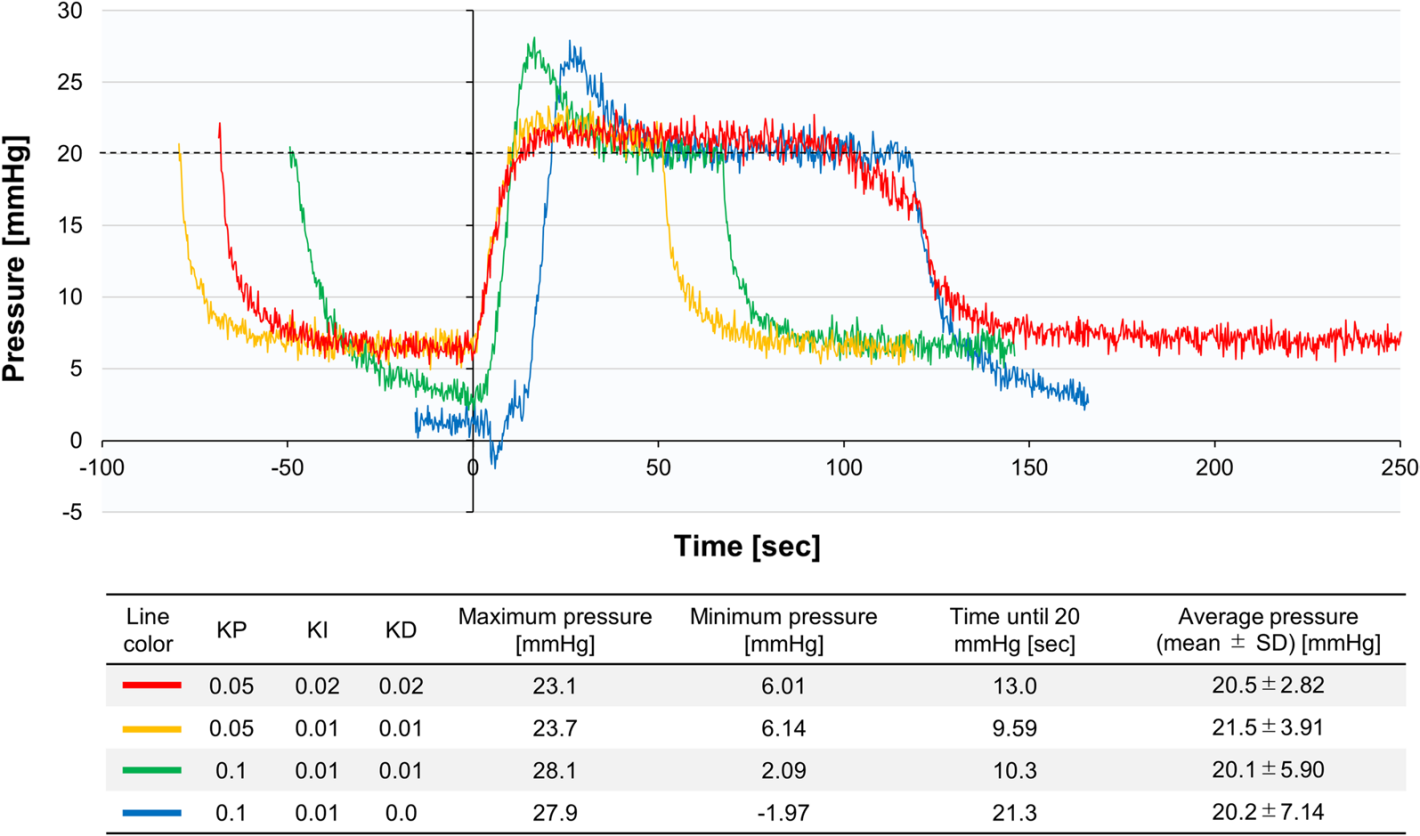
Optimization of PID controller for regulating intrapelvic pressure. Graphs showing the change in pressure based on values of KP, KI, and KD. *PID* proportional–integral–derivative, *SD* standard deviation.

Since the relationship between the rotation speed of the roller pump and IPP in vivo is unknown, the three parameters KP, KI, and KD were determined experimentally. The desired pressure was set to 20 mmHg, and the parameters were adjusted to evaluate the system’s behavior. The maximum rotation speed was set to 50 rpm to avoid excessive pressure. The measurement results are shown in Fig. 4. When comparing KP values of 0.1 and 0.05, there was no significant difference in the rise time. To prevent overshooting, a KP value of 0.05 was applied in our system. To minimize deviations between the desired pressure and the actual pressure, KI and KD were set to 0.02 in the present experiment.

#### Evaluation of the performance of the system

While setting the target renal pelvic pressure to ascend or descend at values of 5, 10, 15, and 20 mmHg, the values of a-IPP, fo-IPP, and the number of rotations of the irrigation pump were recorded (KP = 0.05, KI = 0.02, KD = 0.02). Furthermore, the performance of the system during ureteroscope movement and insertion of a laser fiber into the working channel (KP = 0.5, KI = 0.02, KD = 0.02) was also assessed.

### Statistical analysis

Accuracy was assessed using the mean absolute error (MAE) as follows:

3 $$\mathrm{MAE}=\frac{1}{n}\sum_{i=1}^{n}\left|{\text{a-IPP}}_{i}-{\text{fo-IPP}}_{i}\right|.$$

In the equation, *n* is the number of evaluated time points^21^. Precision was determined by calculating the standard deviation (SD) of the mean of each IPP value. Pearson’s product moment correlation coefficient was measured to evaluate the degree of correlation between two variables. Student’s *t*-test was used for comparing two independent variables. All statistical analyses were performed using EZR version 1.51 (Saitama Medical Center, Jichi, Japan)^22^. A two-sided p value of less than 0.05 was considered to indicate a statistically significant difference.

## Results

### Assessment of a fiber-optic pressure sensor

#### Impact of the fiber-optic pressure sensor on f-URS deflection

**Figure 5:**
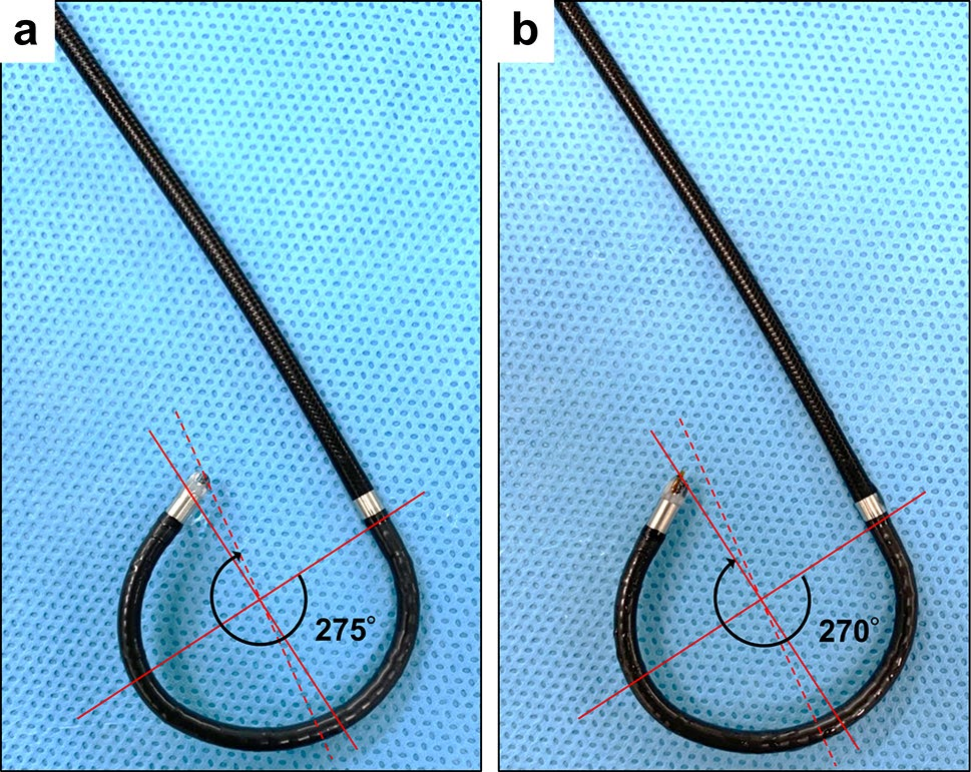
The maximum deflection angle of a flexible ureteroscope. (**a**) An empty working channel. (**b**) A working channel with a fiber-optic pressure sensor.

First, we examined the restriction of the deflection angle when inserting the fiber-optic pressure sensor into the f-URS. The upward and downward maximum deflection angles of the f-URS were both 275°. Placing the fiber-optic pressure sensor into the 3.5-Fr working channel resulted in only 1.8% of lost deflection angle on both sides of deflection, suggesting no significant negative impact on the deflection maneuver during surgery (Fig. 5a,b).

#### Performance of the fiber-optic pressure sensor with usual irrigation pressure

**Figure 6:**
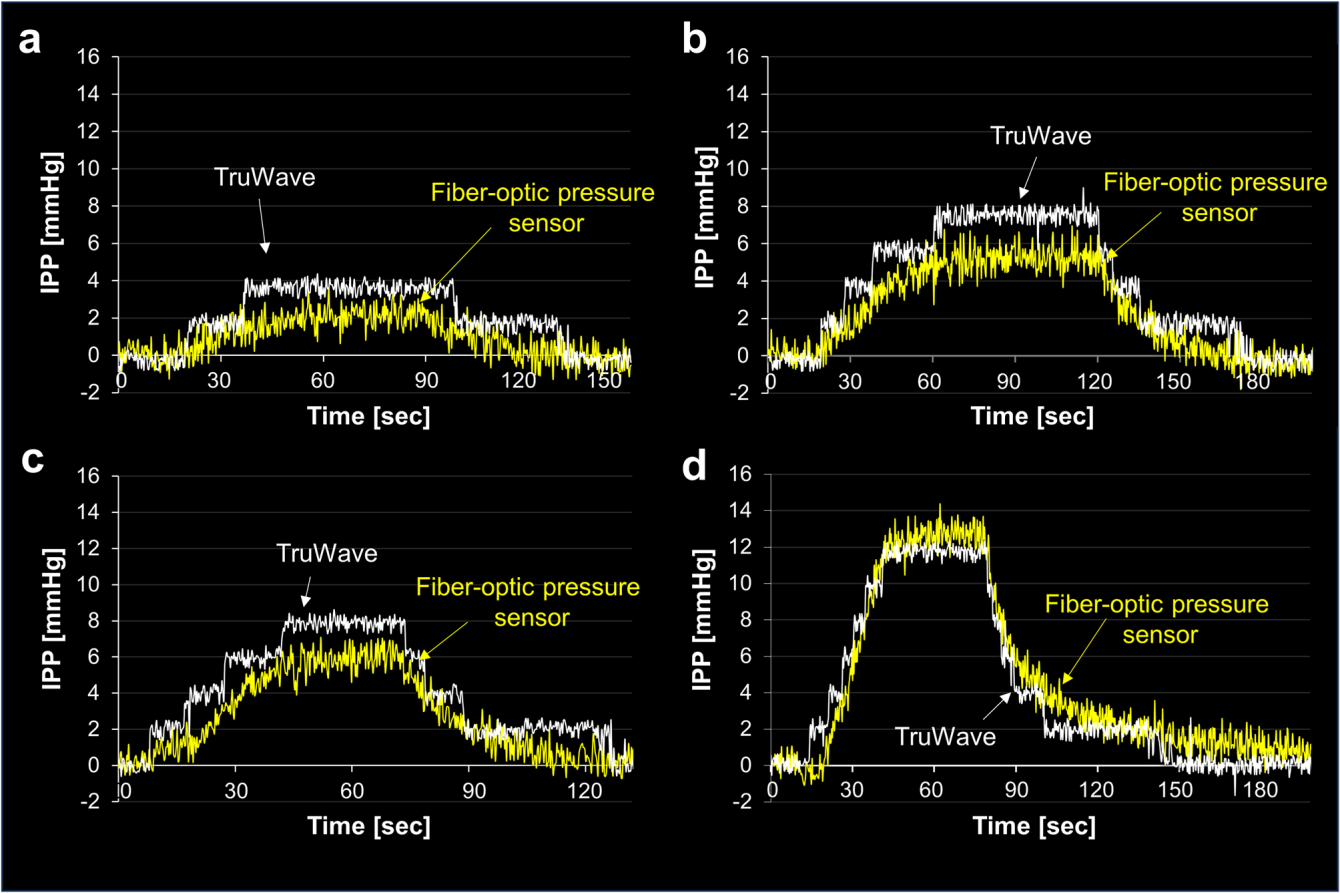
Correlation of intrapelvic pressure (IPP) between the TruWave^®^ transducer and the fiber-optic pressure sensor. Irrigation pressure of (**a**) 60 mbar, (**b**) 100 mbar, (**c**) 120 mbar, and (**d**) 180 mbar.

Next, we examined the measurement accuracy and precision of the fiber-optic pressure sensor in comparison to a-IPP. At an irrigation pressure of 60 mbar during the plateau phase (time of 38.4–97.8 s), fo-IPP and a-IPP showed median values (± SD) of 1.95 ± 0.61 mmHg and 3.62 ± 0.31 mmHg, respectively, with an MAE of 1.66 mmHg (Fig. 6a). The overall correlation coefficient between a-IPP and fo-IPP was 0.76 (95% CI 0.72–0.79%; p < 0.001; Fig. S1a). At an irrigation pressure of 100 mbar during the time of 63.0–199.4 s, fo-IPP and a-IPP were 5.21 ± 0.62 mmHg and 7.53 ± 0.35 mmHg, respectively, with an MAE of 2.31 mmHg (Fig. 6b). The correlation coefficient between the two IPPs was 0.95 (95% CI 0.94–0.95; p < 0.001; Fig. S1b). At an irrigation pressure of 120 mbar during the time of 44.4–73.8 s, fo-IPP and a-IPP were 5.87 ± 0.66 mmHg and 7.86 ± 0.33 mmHg, respectively, with an MAE of 1.92 mmHg (Fig. 6c). The correlation coefficient between the two IPPs was 0.93 (95% CI 0.92–0.94; p < 0.001; Fig. S1c). Furthermore, at a 180-mbar irrigation pressure with a period of 42.0—78.6 s, fo-IPP and a-IPP were 12.56 ± 0.64 mmHg and 11.76 ± 0.30 mmHg, respectively, with an MAE of 0.90 mmHg (Fig. 6d). The correlation coefficient between the two IPPs was 0.97 (95% CI 0.97–0.98; p < 0.001; Fig. S1d).

Overall, the ascending or descending wave shapes of a-IPP appeared as stepped line graphs, whereas the shapes of fo-IPP were smooth line graphs, suggesting that the fiber-optic pressure sensor had a higher resolution compared to the TruWave transducer at low levels of IPP (less than 15 mmHg) (Fig. 6a–d).

#### Pressure dynamics in bolus irrigation

**Figure 7:**
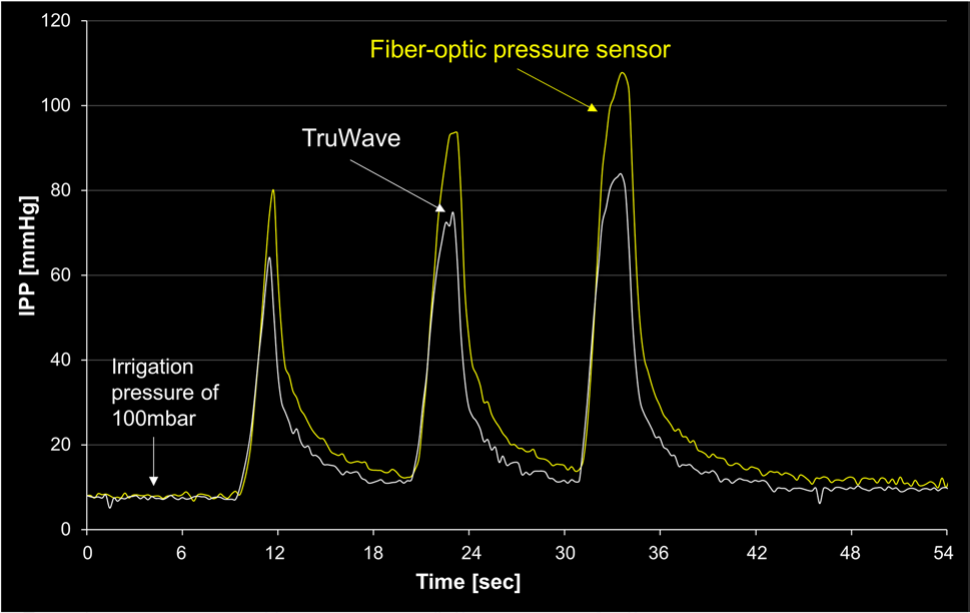
Correlation of intrapelvic pressure (IPP) between the TruWave^®^ transducer and the fiber-optic pressure sensor during force irrigation.

After confirming the stable IPP values (fo-IPP, 7.92 ± 0.42 mmHg; a-IPP, 7.42 ± 0.50 mmHg) with 100 mbar irrigation pressure, bolus irrigations were performed three times (Fig. 7). The mean response time between the fiber-optic pressure sensor and TruWave was comparable (2.96 ± 0.37 s vs. 2.80 ± 0.37 s, respectively; p = 0.613). However, the fiber-optic pressure sensor had a trend toward showing a higher mean peak range than that of TruWave (81.54 ± 11.55 mmHg vs. 62.68 ± 8.67 mmHg; p = 0.0864). The correlation coefficient between the two IPPs was 0.98 overall (95% CI 0.97–0.98; p < 0.001; Fig. S2).

### Development of automatic irrigation system for f-URS

**Figure 8:**
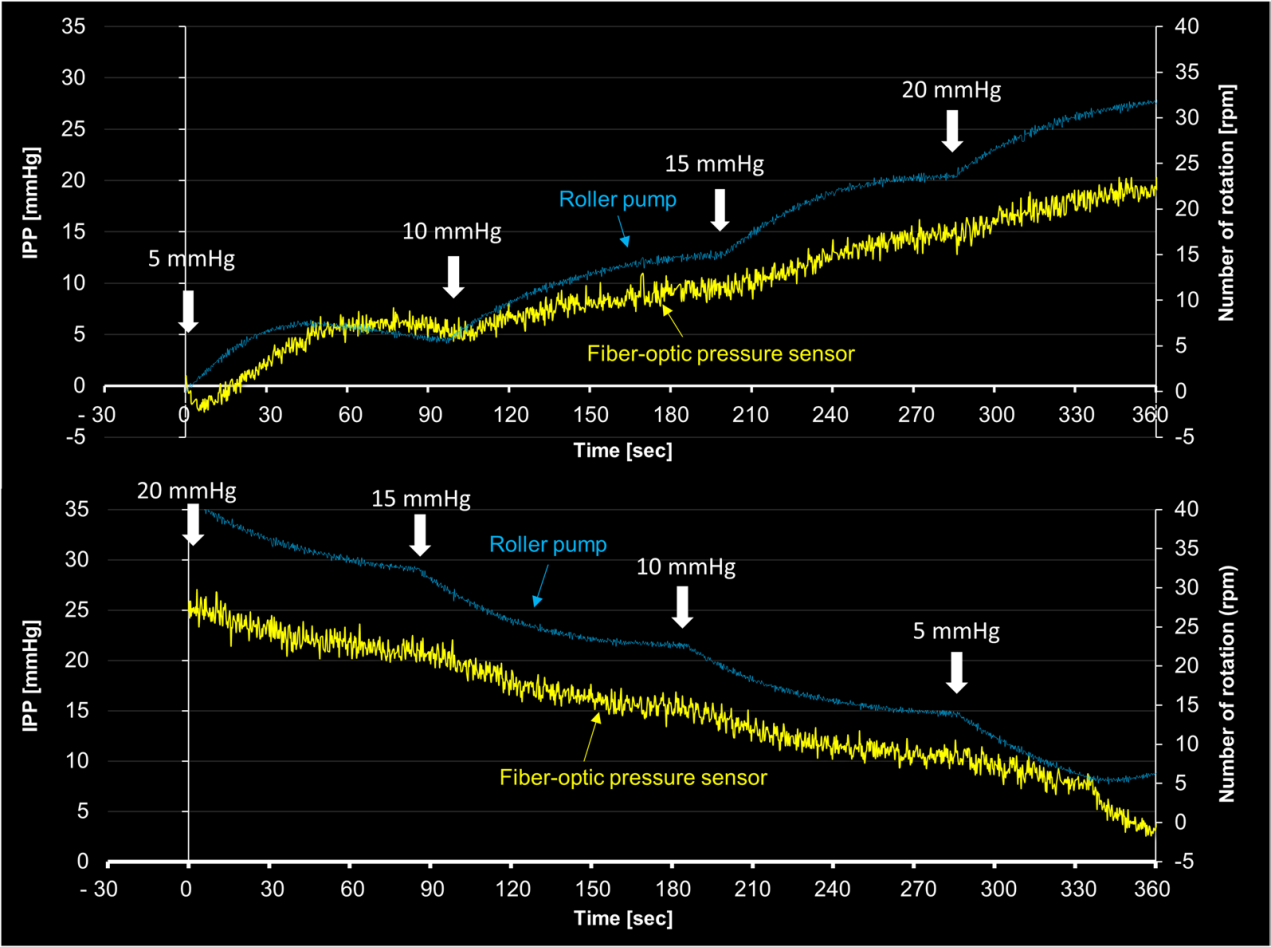
Regulation of intrapelvic pressure (IPP) using a developed automatic irrigation system. Set values input to the PID controller were 5, 10, 15, and 20 mmHg. The vertical axis on the right shows the rotation speed of the roller pump.

After optimization of the PID controller as described in the “Materials and methods” section, we examined whether the automatic irrigation system correctly regulated IPP in the ex vivo kidney model. Desired values were set in order as 5 mmHg, 10 mmHg, 15 mmHg, and 20 mmHg, or in reverse order. While controlling the number of rotations of the irrigation roller pump, this feedback system smoothly and successfully regulated IPP without overshooting according to the set IPP values (Fig. 8). The average time to reach the target pressure was 96.6 ± 6.0 s during the rise process and 85.8 ± 18.6 s during the drop process.

### Performance evaluation of the developed automatic irrigation system for f-URS

#### Impact of movement of f-URS

**Figure 9:**
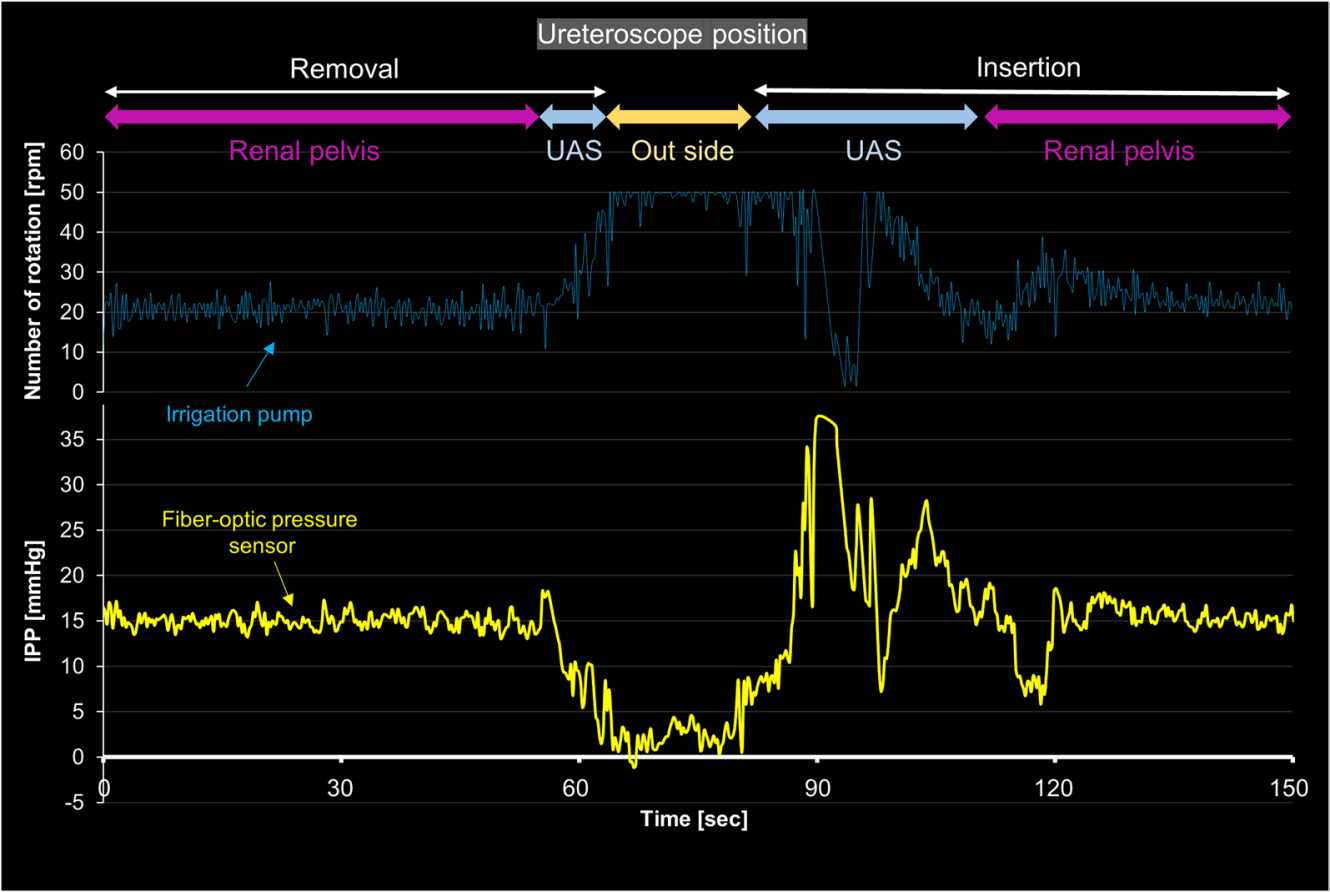
Change in intrapelvic pressure (IPP) and the rotation speed of the roller pump during the insertion and removal of a ureteroscope through a ureteral access sheath (UAS) equipped with the developed automatic regulation system.

We investigated the influence of ureteroscope movement on IPP regulation during the insertion and removal of the ureteroscope through a ureteral access sheath, as depicted in Fig. 9. The irrigation system maintained a preset value of 15 mmHg. When the ureteroscope was removed, a rapid decline in IPP was observed, concomitant with an increase in pump motor rotation speed, reaching 50 rpm (the maximum limit for the roller pump). During ureteroscope insertion, there were occasional rapid fluctuations, similar to noise, caused by air pressure or retained irrigation fluid on the optical fiber pressure sensor. However, once the ureteroscope reached the renal pelvis, there were no instances of overshooting or undershooting, and the pressure promptly stabilized at the desired value of 15 mmHg.

#### Impact of inserting a laser fiber into the working channel

**Figure 10:**
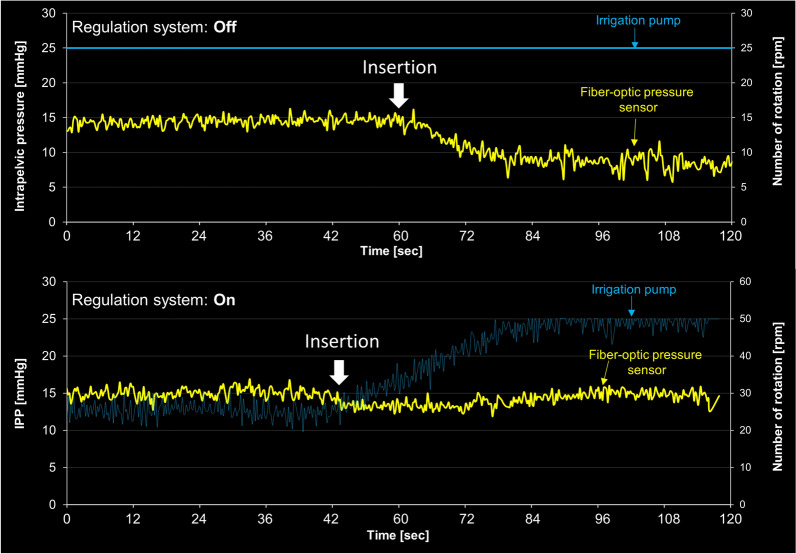
Change in intrapelvic pressure (IPP) during the insertion of a 272 μm laser fiber, with or without the developed automatic regulation system. The vertical axis on the right shows the rotation speed of the roller pump.

We conducted additional experiments to investigate the effect of inserting a 272 μm laser fiber through the working channel on IPP, as demonstrated in Fig. 10. In the absence of a PID controller system, with a fixed pump rotation speed of 25 rpm, IPP gradually decreased from 14.48 ± 0.64 to 8.55 ± 1.03 mmHg. However, when using the regulation system, we observed a slight decrease in IPP immediately after the insertion. The pump rotation speed quickly increased from approximately 25 rpm to 50 rpm, effectively maintaining IPP at the designated value of 15 mmHg.

## Discussion

In the present study, we investigated the utility of a fiber-optic pressure sensor placed on f-URS for evaluating IPP and developed an automatic irrigation system with feedback regulation based on a PID controller. The accuracy of the fiber-optic pressure sensor, compared to the TruWave, showed a MAE of 0.9–2.31 mmHg at irrigation pressures of 60–180 mbar. For precision, SDs of mean pressure values measured by the fiber-optic pressure sensor were 0.61–0.66 mmHg. In forced irrigation, although the response time was equivalent between the two sensors, the peak range of the fiber-optic pressure sensor was higher than that of TruWave, which will be discussed later. Overall, we observed median-to-strong correlations between fo-IPP and a-IPP regardless of irrigation pressures. Finally, after determining the optimal setting of the PID controller, we successfully developed the automatic irrigation system synchronizing IPP monitored by the fiber-optic pressure sensor, which worked smoothly and correctly. Although this system is currently a prototype for clinical use, it holds the potential to facilitate the regulation of safe IPP during ureteroscopic procedures.

Traditionally, pyelovenous backflow at 30 mmHg is usually quoted as a contributing factor to complications from intrarenal surgery, which was described by Hinman^23^. However, little is known concerning the actual IPP value of pyelovenous backflow, because vascular permeability due to the inflammation varies according to the patient’s condition; Hong et al.^13^ reported on 5/37 patients who had postoperative complications, with mean IPPs of 15.2–79.5 mmHg and maximum IPPs of 25.8–310.6 mmHg. Recent studies have demonstrated the utility of a vascular pressure wire, which consists of a fiber-optic pressure sensor and wire staff, to investigate IPP during f-URS^11–14^. However, there has been a lack of studies regarding whether the specifications of commercially available fiber-optic pressure sensors accurately reflect IPP.

This study clarified the specifications of the sensor required for measuring IPP by comparing it to a commonly used conventional pressure transducer, as follows: (i) Although there was a maximum MAE of 2.31 mmHg in accuracy at low IPP, it is presumed to be due to the lower sensitivity of the conventional transducer, as indicated by the stepped graph, compared to that of the fiber-optic pressure sensor. (ii) The conventional transducer was relatively superior in terms of precision; however, the difference of approximately 0.3 mmHg does not pose a clinical problem. (iii) During bolus irrigation, the fiber-optic pressure sensor showed a higher mean value of approximately 18.86 mmHg than the conventional transducer. Additional experiments using our prototype optic-fiber sensors in the situation of bolus irrigation (data not shown) confirmed higher maximum values detected in fo-IPP compared to a-IPP, similar to our initial results. A possible explanation for this is that friction from the nephrostomy tube may contribute to pressure loss, represented by the Darcy–Weisbach formula, Δ p = f (L/d)(ρv^2^/2) [Δ p: pressure loss (m), f: Darcy–Weisbach friction coefficient, L: length of tube (m), d: inner diameter of tube (m), ρ: density of fluid (kg/m^3^), and v: flow velocity (m/s)]. This indicates that pressure loss (Δ p) increases as irrigation fluid velocity (v) increases. Thus, our results suggest that the conventional catheter-based measurement method, which is commonly used in clinical and experimental settings, may underestimate the actual pressure, especially in cases of rapid IPP increase. In this regard, the fiber-optic pressure sensor built-in f-URS, which directly measures IPP, can be considered an ideal tool for clinical practice.

Regarding the automatic irrigation system, a relatively longer time was required to achieve stable IPP when increasing the set pressure compared to decreasing it (96.6 ± 6.0 s vs. 85.8 ± 18.0 s). This might be attributed not only to the gradual increase in drainage via the ureteral access sheath in low IPP but also to the elastic resistance of the renal pelvic wall during the expansion of the renal pelvis as the pump increases the irrigation flow, causing delays in obtaining stable pressure^24^. Conversely, during pressure reduction, the renal pelvic wall is already distended with high IPP, and its elastic force directly contributes to increasing drainage, resulting in a shorter time to reach the target pressure. Moreover, the pressure fluctuation (standard deviation) was more significant during the pressure reduction process, which might be due to the time to reach the target pressure depending on the drainage rate (i.e., higher intrarenal pressure leads to faster drainage, and lower intrarenal pressure results in slower drainage). Thus, an increase in standard deviation during the pressure reduction process might have been observed.

As there is no consensus on the optimal IPP for preventing postoperative complications, a clinical trial is ongoing to elucidate this using an f-URS with the fiber-optic pressure sensor (NCT04900688). If more substantial data clarifies the optimal IPP for different patient characteristics, our developed automatic irrigation system could be applied in clinical practice accordingly. However, there are several limitations of this system that should be addressed before clinical use. First, the feasibility and safety of the automatic irrigation system should be tested using multiple in vivo models with different types of ureteroscope, ureteral access sheath, and surgical setting. Second, the conventional catheter-based measurement method, which is commonly used in clinical and experimental settings, may underestimate the actual pressure, especially during rapid IPP increases. This suggests that a more suitable experimental setup should be explored. Third, it should be evaluated whether a stable operation can be obtained even under harsh surgical maneuvers and conditions. Most importantly, we must evaluate whether this system contributes to the standardization of surgical procedures, regardless of surgeons’ experience.

## Conclusions

We successfully developed an automatic irrigation system using an ultra-miniature fiber-optic pressure sensor for controlling IPP during f-URS. Through optimization of the PID controller, we identified the optimal values and demonstrated the system’s capability to regulate targeted IPPs effectively. Although this system is currently a prototype for preclinical use, its potential impact on endourological devices and standardization of surgical skills holds promise for achieving favorable clinical outcomes.

### Supplementary Information

Supplementary Figures.

## Data Availability

The data that support the findings of this study are available from the corresponding author upon reasonable request.
