# Supplementary material for: Automatic irrigation system with a fiber-optic pressure sensor regulating intrapelvic pressure for flexible ureteroscopy
Source: Sci Rep. 2023 Dec 21;13:22853. doi: 10.1038/s41598-023-47373-5 (PMC10739729; doi:10.1038/s41598-023-47373-5)
Supplement: Supplementary file 1 — Supplementary Figures. [file 41598_2023_47373_MOESM1_ESM.docx]

**Automatic irrigation system with a fiber-optic pressure sensor regulating intrapelvic pressure for flexible ureteroscopy**

Takashi Yoshida, MD, PhD^1, 2^, Noriko Tsuruoka, BE, PhD^3^, Yoichi Haga, MD, PhD^3, 4^, Hidefumi Kinoshita, MD, PhD^2^, Sang-Seok Lee, PhD^5^, and Tadao Matsunaga, BE, PhD^1^

^1^Graduate School of Engineering, Micro- and Nano-device Engineering, Tottori University, Tottori, Tottori, Japan

^2^Department of Urology and Andrology, Kansai Medical University, Osaka, Japan

^3^Graduate School of Engineering, Tohoku University, Sendai, Japan

^4^Graduate School of Biomedical Engineering, Tohoku University, Sendai, Japan

^5^Graduate School of Engineering, Tottori University, Tottori, Tottori, Japan

-1

0

1

2

3

-1

0

1

2

3

4

**fo-IPP [mmHg]**

**a-IPP [mmHg]**

-2

0

2

4

6

0

2

4

6

8

0

2

4

6

0

2

4

6

8

0

5

10

-2

0

2

4

6

8

10

12

**a**

**b**

**c**

**d**

**fo-IPP [mmHg]**

**fo-IPP [mmHg]**

**fo-IPP [mmHg]**

**a-IPP [mmHg]**

**a-IPP [mmHg]**

**a-IPP [mmHg]**

r = 0.76 (95%CI 0.72-0.79)

r = 0.95 (95%CI 0.94-0.95)

r = 0.93 (95%CI 0.92-0.94)

r = 0.97 (95%CI 0.97-0.98)

**Supplementary Fig. 1. Correlation of the actual intrapelvic pressure by TruWave (a-IPP) and the IPP evaluated by the fiber-optic pressure sensor (fo-IPP). Irrigation pressure of (a) 60 mbar, (b) 100 mbar, (c) 120 mbar, and (d) 180 mbar. Pearson's product moment correlation coefficient was used for statistical analysis.**

20

40

60

80

100

20

40

60

80

**Supplementary Fig. 2. Correlation of the actual intrapelvic pressure by TruWave (a-IPP) and the IPP evaluated by the fiber-optic pressure sensor (fo-IPP) during bolus irrigation. Pearson's product moment correlation coefficient was used for statistical analysis.**

**fo-IPP [mmHg]**

**a-IPP [mmHg]**

r = 0.98 (95%CI 0.97-0.98, p<0.001)
